# Epidemiological Algorithm for Early Detection of COVID-19 Cases in a Mexican Oncologic Center

**DOI:** 10.3390/healthcare10030462

**Published:** 2022-03-01

**Authors:** Moisés González-Escamilla, Diana Cristina Pérez-Ibave, Carlos Horacio Burciaga-Flores, Vanessa Natali Ortiz-Murillo, Genaro A. Ramírez-Correa, Patricia Rodríguez-Niño, Rafael Piñeiro-Retif, Hazyadee Frecia Rodríguez-Gutiérrez, Fernando Alcorta-Nuñez, Juan Francisco González-Guerrero, Oscar Vidal-Gutiérrez, María Lourdes Garza-Rodríguez

**Affiliations:** 1 Centro Universitario Contra el Cáncer, Universidad Autónoma de Nuevo León, Hospital Universitario “Dr. José Eleuterio González”, Av. Francisco I. Madero S/N, Mitras Centro, Monterrey 64460, Mexico; drmoisesgzz87@gmail.com (M.G.-E.); dperezi@uanl.edu.mx (D.C.P.-I.); carlos.hburciaga@gmail.com (C.H.B.-F.); patriciardzn@hotmail.com (P.R.-N.); rpineiror@uanl.edu.mx (R.P.-R.); hazyadee@gmail.com (H.F.R.-G.); fernando.alcortanz@gmail.com (F.A.-N.); juan.gonzalezgrr@uanl.edu.mx (J.F.G.-G.); oscar.vidalgtz@uanl.edu.mx (O.V.-G.); 2 Facultad de Medicina, Universidad Autónoma de Nuevo León, Av. Francisco I. Madero S/N, Mitras Centro Monterrey, Monterrey 64460, Mexico; vanessa.ortizmu@uanl.edu.mx; 3 Department of Molecular Science, The University of Texas Rio Grande Valley School of Medicine, McAllen, TX 78504, USA; genaro.ramirezcorrea@utrgv.edu; 4 Department of Pediatrics, Division of Cardiology, Johns Hopkins University School of Medicine, Baltimore, MD 21205, USA

**Keywords:** COVID-19, SARS-CoV-2, prevention, electronic early detection tools

## Abstract

An early detection tool for latent COVID-19 infections in oncology staff and patients is essential to prevent outbreaks in a cancer center. (1) Background: In this study, we developed and implemented two early detection tools for the radiotherapy area to identify COVID-19 cases opportunely. (2) Methods: Staff and patients answered a questionnaire (electronic and paper surveys, respectively) with clinical and epidemiological information. The data were collected through two online survey tools: Real-Time Tracking (R-Track) and Summary of Factors (S-Facts). Cut-off values were established according to the algorithm models. SARS-CoV-2 qRT-PCR tests confirmed the positive algorithms individuals. (3) Results: Oncology staff members (*n* = 142) were tested, and 14% (*n* = 20) were positives for the R-Track algorithm; 75% (*n* = 15) were qRT-PCR positive. The S-Facts Algorithm identified 7.75% (*n* = 11) positive oncology staff members, and 81.82% (*n* = 9) were qRT-PCR positive. Oncology patients (*n* = 369) were evaluated, and 1.36% (*n* = 5) were positive for the Algorithm used. The five patients (100%) were confirmed by qRT-PCR. (4) Conclusions: The proposed early detection tools have proved to be a low-cost and efficient tool in a country where qRT-PCR tests and vaccines are insufficient for the population.

## 1. Introduction

The outbreak of the coronavirus disease 2019 (COVID-19) was declared a pandemic on 11 March 2020 by the World Health Organization (WHO) [1]. The new coronavirus (SARS-CoV-2) can infect people of all ages. However, older people and individuals with pre-existing medical conditions (such as cancer, asthma, diabetes, obesity, hypertension, and heart disease) appear more vulnerable to becoming seriously ill. COVID-19 has a mortality rate >8% in people older than 70 years [2,3].

Oncologic patients are at higher risk of severe COVID-19 infection and worse prognosis than non-cancer patients [2]. Some systematic reviews concluded an increased risk of severe COVID-19 in oncologic patients due to associated co-morbidities linked to cancer [4].

The clinical diagnosis of COVID-19 is based mainly on epidemiological history and clinical manifestations. However, the clinical symptoms and signs of patients infected with SARS-CoV-2 are highly variable. Therefore, additional tests for detecting the virus are needed, such as qRT-PCR [5]. Many people have flu-like symptoms onset; However, widespread population testing is not available in developing countries. Thus, it is essential to develop an early detection tool to evaluate symptoms with the most predictive value to identify COVID-19 without molecular testing. The early detection tool implementation is an effort to guide public health recommendations for self-detection, self-isolation, early detection, and preventing the further spread of the disease.

Digital health and information technologies have emerged to support public health policies and offer opportunities to reshape current health care systems, especially in developed countries [6,7]. They are used for early detection, prevention, and advice for skeletal injuries, chronic orthopedic conditions, smoking, cancer, diabetes, and infectious diseases [8,9,10,11]. Furthermore, digital health and information technologies are helpful for the widespread distribution of information, real-time COVID-19 transmission tracking, virtual venue creation, and telemedicine visits for patients [12,13,14,15,16,17,18]. Digital health and information technologies are helpful in infectious diseases such as HIV, bacterial resistance, and hospital sepsis.

In Mexico, qRT-PCR tests are not available for the entire population. Since the virus began spreading in the country, nearly 2500 Mexican health workers have died from COVID-19, and 188,000 have been infected as of 4 January 2021. Mexico holds the highest mortality rate in patients and medical staff worldwide [19]. Therefore, digital technologies’ early detection tools can be an excellent option for detecting and preventing COVID-19 in our country.

As a cancer center in a developing country that works with high-risk patients, this study aimed to develop and implement two early detection tools that used self-reported COVID-19 symptoms for patients and oncology staff members (O.S.M.) for early diagnosis at low cost. For validation of these two algorithms, we calculated the sensitivity for each detection algorithm for COVID-19 positive cases confirming results by qRT-PCR and immunological test. In addition to this, we implement personal protective equipment, isolation measures, staff training, and sanitary fences for SARS-CoV-2.

## 2. Materials and Methods

### 2.1. Study Population

We used a total of 142 O.S.M. and 369 patients. We conducted a retrospective research study in the Centro Universitario Contra el Cáncer (C.U.C.C.) of the Universidad Autónoma de Nuevo León (U.A.N.L.) in Monterrey, Nuevo León, México. The work areas of the oncology staff include medical oncology, radiotherapy (R.T.), nursing, molecular biology laboratory, social work, C.U.C.C. Early Cancer Detection Clinic (C.E.C.I.L.), clinical protocols area, and administration. Patients were visited in the Radiotherapy Unit of C.E.C.I.L. This study was conducted following the Declaration of Helsinki. The Institutional Ethics Committee approved the protocol of the University Hospital (ON21-00012). All employees and patients answered a questionnaire with clinical and epidemiological information, and a confirmatory molecular test was applied to those who presented suggestive COVID-19 symptoms.

### 2.2. O.S.M. Training and Personal Protection Equipment (P.P.E.)

All O.S.M. were trained to prevent and identify SARS-CoV-2 infection. A face-to-face course was held, keeping a 6 ft. distance. Groups of 10 people received training from a radio-oncologist, a nurse, and two PhDs. The course lasted one hour and aimed to educate oncology staff to identify the most common symptoms for both cancer and non-cancer patients and the period of appearance of symptoms. They also received training to answer an electronic survey with two predictive models for the early detection of probable cases of COVID-19; they were given the correct filling steps, the link, and the Q.R. code to answer the survey. The sanitary fences (isolation) assigned to prevent the transmission of the virus were identified; the importance of handwashing and the use of a face mask were explained. The steps for sanitizing areas and how to prepare the cleaning solutions were also described.

Individual safety measures were implemented for all O.S.M., among which the use of P.P.E. (face masks and face shields) was mandatory and provided by the Oncology Center. COVID-19 symptomatic O.S.M. were treated at U.A.N.L. Employee Health Clinic, and severe symptomatic O.S.M. were referred to the COVID-19 U.A.N.L. Hospital.

### 2.3. Application of Electronic Surveys to O.S.M.

The O.S.M. was given the “Survey for Personnel-Test Covid 19” from March 2020 to January 2021, with a frequency of 2 electronic surveys per week. Two predictive algorithm models were used: Real-Time Tracking (R-Track) and Summation of Factors (S-Facts). The surveys were applied through mobile devices to monitor symptoms in the staff and detect potential COVID-19 cases before entry to the work area. Employee’s positives for the algorithms were confirmed by qRT-PCR. In addition to the algorithm, epidemiological variables, nonspecific (general) symptoms, co-morbidities, and risk factors were documented. We also include questions to trace O.S.M.’s close contact with COVID-19 positive individuals, such as family members or friends with symptoms or confirmed with COVID-19. No person with fever or low oxygen saturation, including O.S.M. or patients, were admitted to ingress to C.U.C.C. They were referred directly to the COVID-19 U.A.N.L. Hospital for clinical evaluation and qRT-PCR tests.

#### 2.3.1. R-Track Predictive Model

The R-Track survey was adjusted for age, sex, and symptoms (dysgeusia and/or anosmia, persistent dry cough, severe fatigue, and meal skipping) to identify symptoms correlated with COVID-19. The following formula was used to identify the probable COVID-19 cases:

x = −1.32 (0.01 × Age) + (0.44 × Sex) + (1.75 × dysgeusia and/or anosmia) + (0.31 × Persis-tent dry cough) + (0.49 × Severe fatigue) + (0.39 × Meal skipping).

A value of 1 was assigned if the person self-reports the symptom and 0 if not. The sex feature was also binary, with 1 indicative of male participants and 0 representing females. The values were transformed into predictive probability using exp(x)/(1  +  exp(x)). A result calculation followed by assigning cases of predicted COVID-19 for probabilities ≥0.5 and controls for probabilities <0.5, according to the Menni y cols study [13].

#### 2.3.2. S-Facts Predictive Model

In the S-Facts survey, the most common and specific symptoms of the disease were evaluated. The formula used was the following:

Probability = (1.0 ×Dyspnea) + (1.0 × Temperature ≥ 38.0 °C) + (1.0 × Chest pain) + (1.0 × Dysgeusia and/or anosmia) + (1.0 × Persistent dry cough) + (1.0 × Severe fatigue) + (1.0 × Meal skipping).

Results with values ≥3.0 were considered positive for SARS-CoV-2 risk infection [14].

### 2.4. Detection of SARS-CoV-2 IgM and IgG Antibodies in O.S.M.

A total of 140 O.S.M. were tested for COVID-19 antibodies in March 2021. We used the qualitative membrane-based immunoassay Coronavirus (2019-nCoV) IgG/IgM Test Kit (Genrui^®^ Biotech Inc., Shenzhen, China) to detect IgM and IgG antibodies against SARS-CoV-2 in whole blood according to the manufacturer’s instructions.

### 2.5. Paper Surveys for Radiotherapy Area Patients

A paper survey was applied to cancer patients treated at the radiotherapy area from October 2020 to February 2021. Temperature and oxygen saturation were recorded before patients attended at C.E.C.I.L. clinic. The survey studied the most significant symptoms for COVID-19 (temperature ≥ 38 °C, persistent dry cough, dyspnea, dysgeusia, and/or anosmia). Nonspecific symptoms such as headache, diarrhea, chest pain, runny nose, arthralgia, myalgia, redness, and itching eyes, were also registered.

Radiotherapy patients had additional screening questions. Further questions included: (1) If they had direct contact with a confirmed COVID-19 positive family member or friend, (2) If they traveled outside the Monterrey metropolitan area in the last 15 days (Supplementary Material 1: Table S1). A value ≥7 indicated a high probability of having COVID-19 (positives for the algorithm). Patients were confirmed by qRT-PCR.

Additionally, we designed a unique brochure for oncology patients. All patients and their caregivers who entered the C.U.C.C. for consultation, chemotherapy, radiotherapy, or consultation received the pamphlet. The pamphlet emphasizes the importance of early detection and prevention of COVID-19 infection in oncological patients. It also details the clinical data presented in cancer patients and the high risk of developing severe symptoms (Supplementary Material 2: Figure S1).

### 2.6. Sample Collection for SARS-CoV-2 qPCR Assay

The samples were collected following the CDC (Control Disease Center) guidelines [15]. The detailed information on packing, shipping, and transporting specimens can be found at Interim Laboratory Biosafety Guidelines for Handling and Processing Specimens Associated with Coronavirus Disease 2019 (COVID-19) on the CDC website [15].

### 2.7. RNA Extraction

After nasopharyngeal sample collection, total RNA was isolated using the QIAamp^®^ RNA Viral Mini Kit from QIAGEN (Hilden, Germany) in the QIAcube connect system from QIAGEN according to the manufacturer’s instructions. The RNA samples were stored at −80 °C until further use.

### 2.8. qRT-PCR Tests

We used qRT-PCR assay 2019-nCoV R.N.A. (2019-nCoV) Probe Assay from IDT (Integrated DNA Technologies Inc., Coralville, IA, USA). Two targets from one gene, the nucleocapsid protein (2019-nCoV_N1 and 2019-nCoV_N2) and the human gene RNAase, were simultaneously amplified during the real-time RT-PCR assay. These diagnostic conditions follow the CDC recommendations conditions.

## 3. Results

### 3.1. Electronic Surveys and Antibodies Detection in O.S.M.

**Figure 1:**
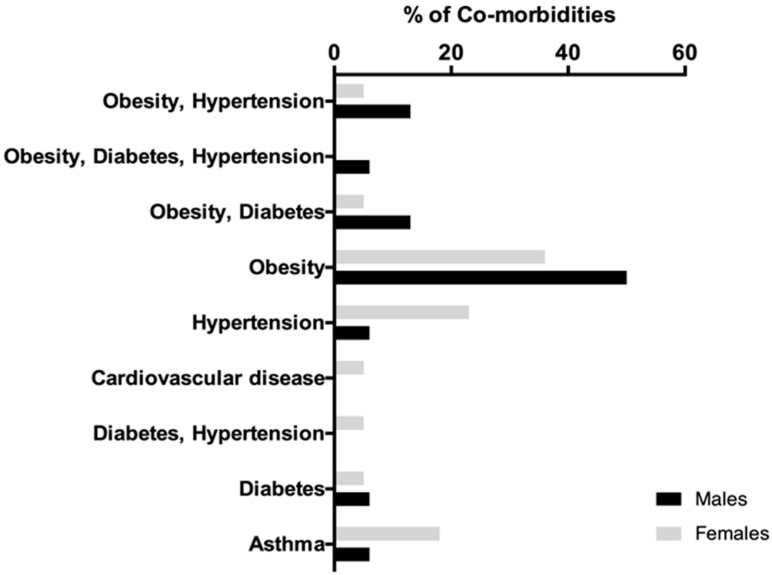
Representation of frequency for the most common co-morbidities present in the O.S.M. (*n* = 38) in an oncology center. This graph shows in percentage (%) the frequency of each different co-morbidities; men are represented by a solid black bar and women by a solid gray bar.

O.S.M. were surveyed (*n* = 142, men *n*= 58 and women *n* = 84, median age 32 years old, range of 18–67 years old). Two surveys a week yielded a total of 3327 surveys from March 2020 to January 2021. We also studied the presence of the most relevant risk co-morbidities for COVID-19 in O.S.M. and patients. We found that 26.8% of employees had co-morbidities (*n* = 38, men *n* = 16, and women *n* = 22). Obesity was the more frequently observed co-morbidity in men 50% (8/16) and women 36.4% (8/22). Hypertension in 22.7% (5/22) and asthma in 18.2% (4/22) were among the most common (Figure 1).

#### 3.1.1. R-Track and S-Facts Algorithm for O.S.M Screening

With the R-Track model, we identified 20 employees with suggestive clinical data of COVID-19 infection, of which 75.0% (15/20) were qRT-PCR positive for SARS-CoV-2.

Using the S-Facts Algorithm, we identified 11 employees with suggestive symptoms of COVID-19, of which 81.8% (9/11) were qRT-PCR positive. False-positive results were obtained with both models. With the R-Track model, five employees presented alerts in the algorithm. Still, they were qRT-PCR negative, and the S-Fact two employees with a warning in the algorithm were qRT-PCR negative.

A total of 12% (17/142) of O.M.S. identified as positive with the algorithms were SARS-CoV-2 qRT-PCR positive. The age range was 18 to 62 years, and 58.8% (10/17) were female. Among positive O.S.M., obesity was observed in 23.5% (4/17). Administrative and nursing staff had the highest incidence of COVID-19 with 35.3% (6/17) per group (Table 1). We found that 64.7% (11/17) of staff with SARS-CoV-2 qRT-PCR positive also documented a high-risk exposure. Close contact with individuals with symptoms or previous COVID-19 infection increased the probability of contracting the disease (Table 1).

We analyzed the main symptoms of the oncology staff with COVID-19. We found that the most common symptoms were skipped meals with 76.5% (13/17) and dysgeusia/anosmia with 82.4% (14/17). Other symptoms included headaches with 70.6% (12/17), muscle or body aches with 70.6% (12/17), runny nose with 58.8% (10/17), and sore throat with 58.8% (10/17) (Table 1).

**Figure 2:**
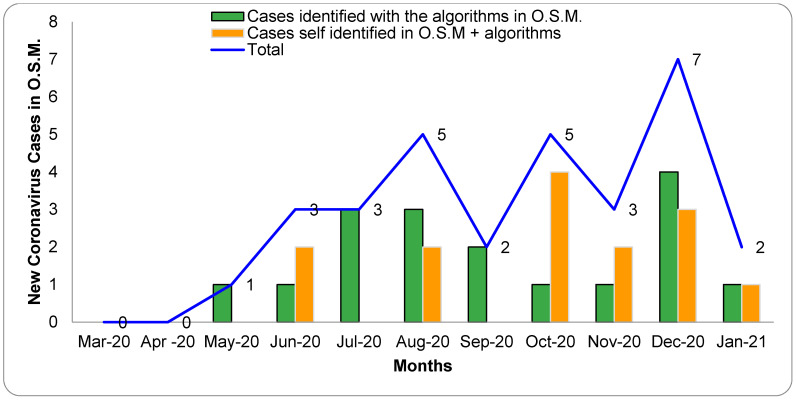
Representation of COVID-19 diagnosed cases in 2020 in the oncology staff members (O.S.M.). This graph shows the number of new cases of COVID-19 per month from March 2020 to January 2021. The employees that were positive with the algorithms (S-Facts and R-Track) are represented by a solid green bar and in a solid orange in bar the self-reported O.S.M. cases (employees with suggestive symptoms for COVID-19 and decide to do PCR test and also respond the algorithms). The blue line represents the total of new COVID-19 cases per month. All the employees with positive algorithm results were confirmed for the SARS-CoV-2 qRT-PCR test.

O.S.M. (*n* = 14) that self-identified suggestive symptoms of COVID-19 directly visited the COVID-19 UANL hospital. All were confirmed the COVID-19 diagnosis with a positive qRT-PCR test. In May 2020, the first oncology staff member was confirmed with COVID-19. The month with the highest incidence of COVID-19 infection was December, where we found seven new confirmed cases in total (cases identified with/without the algorithms). In total, 21.8% (31/142) of O.S.M. were qRT-PCR SARS-CoV-2 positive (Figure 2).

#### 3.1.2. Identification of Antibodies in O.S.M.

Antibody detection frequency was estimated in March 2021 on 140 O.S.M. We found that 44.3% (62/140) of the working population gave positive to the rapid test for antibodies (Table 2). Antibodies against COVID-19 were detected in 7.9% (11/140) of O.S.M. with no previous infection or vaccine against COVID-19. Therefore, a modest fraction of O.S.M. had an asymptomatic COVID-19 infection (Table 2).

**Figure 3:**
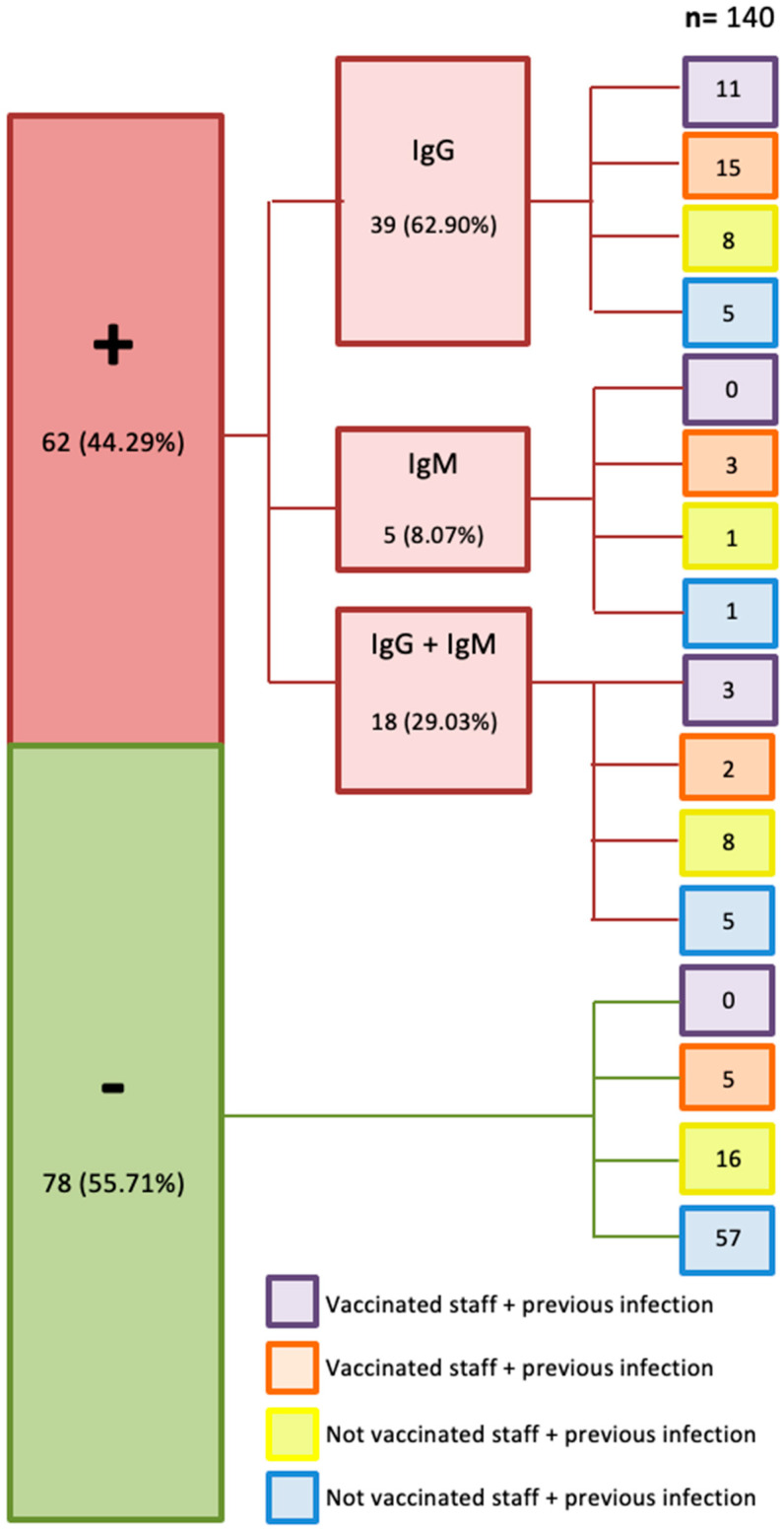
The following representation shows the prevalence of IgM and IgG antibodies for SARS-CoV-2 in the O.S.M. of the cancer center. The big green box represents staff members that do not present antibodies for COVID-19 (55.71%), and the big red box represents staff members that present COVID-19 antibodies (44.29%), which were divided into those that showed antibodies type IgM (8.07%), IgG (62.9%) or both (29.03%). The medical personnel (whether they were positive or negative for COVID-19 antibodies) were in turn divided into personnel who received the vaccine or had a previous infection.

Of the 62 employees who raised antibodies against COVID-19, 62.9% (39/62) generated IgG-type memory antibodies), 28.2% (11/39) had antibodies against COVID-19 after a previous infection, and a COVID-19 vaccine, 25.6% (15/39) had antibodies against COVID-19 after a COVID-19 vaccine without previous infection. We observed that 8.1% (5/62) of O.S.M. had recent infection antibodies (IgM) (Figure 3).

We validated the questionnaire in this O.S.M. population. A total of 91% (10/11) had an asymptomatic COVID-19 infection, 9% (1/11) had minor symptoms (chest pain and dyspnea) that were overlooked by the affected O.S.M. (Table 2), and 27.2% (3/11) reported being previously in contact with positive COVID-19 patients.

### 3.2. Application of Paper Surveys for Patients in the Radiotherapy Area

A total of 4,196 surveys answered by 369 patients were obtained from October 2020 to February 2021. We observed that the average oxygen saturation upon entering the facilities was 96%, and the average temperature was 36.8 °C. We were able to identify five (1.4%) patients with a maximum value of the cut-off point of the S-Facts patient survey, which indicates a high probability of presenting the disease. All five patients underwent a SARS-CoV-2 qRT-PCR confirmation study, and all were positive.

## 4. Discussion

We tested epidemiological algorithms (R-Track and S-Fact) in the field as a paper and electronic survey to identify patients and staff in the radiotherapy area, respectively. We concluded that the sensitivity of the R-Track was 75%, whereas S-Fact was 81.82%. The difference between the model’s surveys efficiency is likely due to COVID-19 symptoms adjusted for age and weight in the R-Track model. The sensitivity of R-Track and S-Fact was slightly higher than Menni et al.’s model, with a sensitivity of 66% and a specificity of 83% [13]. However, the actual specificity of R-Track and S-Fact could not be calculated because the confirmatory test (qRT-PCR) was not performed on all 140 oncology staff members without symptoms.

Another measure that was implemented was the restriction to enter the oncology center for those accompanying cancer patients. To date, only one companion is allowed to enter, only if necessary, according to the patient’s conditions.

Data from ASCO (American Society of Clinical Oncology) supports a higher prevalence of COVID-19 in oncologic patients and a higher risk of death. Especially in leukemia patients and thyroid cancer (odd risk ratio OR of 12.6 and 3.10 respectively). According to ASCO data, the rest of the cancers reported an increased range of odds risk ratio of COVID-19 prevalence (OR of 4.70 to 8.54) and an increased odds risk ratio of death (OR 1.66–3.16) [16,17,18]. Due to the high risk that cancer patients represent, it is of great importance to implement prevention measures. The proposed algorithms represent a low-cost early detection tool.

New SARS-CoV19 variants are reported worldwide. Some of these mutations could have an impact on the immunological response and, in consequence, on the patient’s symptoms. These mutations also can change virulence and the pathogenicity of the virus. For instance, changes in symptomatology could require modifications in the algorithms and prevent programs from ensuring that new cases are identified. Furthermore, changes in the disease duration could require changes in the isolation periods will be required. That is why it is important for the constant evaluation and remodeling of the proposed algorithm [19].

At the time of writing this article, we compared the prevention actions that we implement in our cancer center and others that have been implemented in cancer centers in the USA (United States of America). Our prevention measures are very similar to those implemented in other centers in developed countries, such as the MD Anderson Cancer Center (MDA) [20], the Memorial Sloan Kettering Cancer Center (MSK) [18], and the Dana-Farber Cancer Center (DF) [21]. Among some of the world’s top-ranked oncology centers (MDA, MSK, and DF), in addition to the international prevention guidelines, each one has a specific COVID-19 prevention program displayed on their respective website [16,22].

Due to the training that was carried out on 100% of the oncology staff, a total of 14 employees who went on vacation and/or self-reported having COVID-19 symptoms were not allowed to enter the center. These O.S.M. assisted a COVID hospital, where the PCR-SARS-CoV-2 test was carried out. The period of disability was of two weeks before return to work activities. We think that the self-reported by the O.S.M. is attributed to the training received, with which we provide them the necessary tools for the correct identification of suggestive symptoms of COVID-19. This helped us to maintain a low incidence of infection and thus provide a safe work environment for both employees and patients. No outbreaks were recorded at the C.U.C.C. from March 2020 to January 2021, indicating that the use of electronic surveys, P.P.E., early detection, and preventive training effectively avoided infection outbreaks in our cancer center [23]. Additionally, O.S.M. increased their sense of safety, even in the worst part of the pandemic, because of the intense campaign of symptoms information and self-protection, reinforcing the strict use of the P.P.E. and molecular testing after any suspicious symptoms.

In our study, we observed that dysgeusia/anosmia (82.4%) and meals skipping (76.5%) are the main algorithm symptoms in COVID-19 positive employees, which corresponds to the reported literature [13,24]. Other studies found that fatigue and gastrointestinal manifestations are signs of severe COVID-19 infection [25,26]. We agree that the symptoms presented during the disease correspond to symptoms of good prognosis, and the symptom of diarrhea/vomiting and fatigue was presented in six (35.3%) and five (29.4%) individuals, respectively [27]. None of the employees required hospitalization, for which we conclude that these symptoms are of a good prognosis. However, to confirm this conclusion, a study with a higher number of patients would be required.

Applying a paper survey for all patients admitted to the facilities avoided an outbreak within the radiotherapy area. This approach helped us maintain the patients’ integrity due to the high likelihood of COVID-19 complications in cancer patients [25].

Only 1.3% of the radiotherapy patients presented an alert of possible infection, which indicates a low probability of contracting the disease when receiving cancer treatment within the facilities. We think that using the COVID-19 information pamphlet in cancer patients was of great help for patients to opportune prevent infection [28].

In this study, we detect SARS-CoV-2 IgM and IgG seroprevalence in 44.3% (*n* = 62) of O.S.M. In contrast, Fuereder et al. 2020 reported only 3.2% of seroprevalence in oncology healthcare professionals (from a population of 62 subjects) [29]. Mahto et al. 2021 [30] described 13% of the medical staff with IgG antibodies. The lowest seroprevalence reported was 1.8% in staff members at a French cancer center (from a total of 663) [31]. Published series from Europe in the first wave of the pandemic reported 5–10% of COVID-19 seroprevalence rates [31,32].

From the 11 asymptomatic O.S.M., three reported close contact with family, friends, or patients with symptoms or confirmed COVID-19 infection, and of the remaining nine, the reason for a possible infection is unknown. The incidence of asymptomatic cases reported in different studies varies widely between 1.6% and >50%, depending on the general testing rates and the methodology employed. There is consensus that asymptomatic patients can spread the disease [29,30,32,33]. Additionally, it was demonstrated that asymptomatic individuals exhibited a weaker immune response and had lower levels of neutralizing IgG antibodies [34]. Asymptomatic patients presenting low levels of antibodies might be more likely to become seronegative [35]. Based on these results, we recommend prolonging public health interventions, including social distancing, hygiene, isolation of high-risk groups, and widespread testing.

Within the limitations of the study, it is important to mention that we have limited PCR tests, so we could not perform the PCR on the subjects who were negative for the algorithm. We could not detect if there were asymptomatic subjects, and we did not were able to calculate de specificity of the algorithm. Added to this, we consider that it is necessary to increase the sample size. Moreover, the algorithm does not consider the symptoms associated with the new variants, the immunity acquired through vaccination, reinfections, etc. Therefore, it is necessary to adapt the algorithm considering all these factors.

The mobile market in the developing world is considered to be the most rapidly growing sector [36]. As a perspective, mobile applications and new algorithms will be necessary as new diseases emerge and genome viruses evolve. Our study represents an effective and accessible way to solve many issues in the healthcare sector of the developing world, such as monitoring epidemic diseases as COVID-19. By undertaking these kinds of health tools, the progress of these diseases can be delayed or stopped, the financial cost of the diseases can be reduced, and the quality of patient life can be improved. Although several countries in developing countries, such as Mexico, have limited resources (for tests and vaccines). These countries can employ these tools to improve the quality of healthcare services.

## 5. Conclusions

The use of epidemiological algorithms, as paper and electronic survey tools, in oncology staff and patients provided a low-cost, safe, and practical way to detect COVID-19 to act early on isolation and medical treatment. In this way, we reduced outbreaks in the oncology cancer center. These algorithms applied to oncologic patients are of exceptional value when early detection, prevention and early intervention, and susceptible patients’ outcomes. The proposed algorithms could prove to become a suitable detection tool in countries where qPCR tests and vaccines are insufficient or not available for all the population.

## 6. Patents

Nothing to declare.

## Figures and Tables

**Table 1:** Epidemiological characteristics of 17 O.M.S./qRT-PCR positive for SARS-CoV-2 identified with the algorithms (*n* = 17).

Characteristics	*n* (%)
Gender	
Male	7 (41.18)
Female	10 (58.82)
Co- morbidities	
Obesity	4 (23.53)
Diabetes, Hypertension	1 (5.88)
Any	12 (70.59)
CUCC Work area	
Nursery	6 (35.29)
Interns/Residents	1 (5.88)
Administration	6 (35.29)
Radiation therapist	4 (23.53)
Symptoms	
Fatigue *	6 (35.29)
Skipped meals *	13 (76.47)
Loss of taste/smell *	14 (82.35)
Cough *	9 (52.94)
Fever *	8 (47.06)
Shortness of breath *	4 (23.53)
Chest pain	6 (35.29)
Diarrhea or vomiting	5 (29.41)
Sore throat	10 (58.82)
Pink eyes	3 (17.65)
Runny nose	10 (58.82)
Muscle or body aches	12 (70.59)
Headaches	12 (70.59)
Risk exposure	
Exposure with COVID-19 positive patients	11 (64.71)
Exposure with family/friends with symptoms of COVID-19	9 (52.94)
Exposure with positive family/friends COVID-19	10 (58.82)

* Symptoms that are directly evaluated with the algorithms.

**Table 2:** Results of the rapid antibody test in the O.S.M. (*n* = 140).

Antibody Test	Oncology Staff *n* = 140 (%)	Vaccinated	With Previous Infection	Vaccinated + Previous Infection	Non Vaccinated without Previous Infection
Positive	62 (45%)	20	17	14	11
Negative	78 (57%)	5	16	0	57

## Data Availability

Does not apply.

## Supplementary Materials

The following Supplementary Material was included in https://www.mdpi.com/article/10.3390/healthcare10030462/s1; Table S1: Questionary survey for Radiotherapy area patients; Figure S1: Brochure with information on the covid-19 for cancer patients at the center (this file is in Spanish).
