# Supplementary material for: Epidemiological Algorithm for Early Detection of COVID-19 Cases in a Mexican Oncologic Center
Source: Healthcare (Basel). 2022 Mar 1;10(3):462. doi: 10.3390/healthcare10030462 (PMC8950794; doi:10.3390/healthcare10030462)

LIBAC
LABORATORIO DE INVESTIGACIÓN BÁSICA-CLÍNICA

Table S1

CENTRO UNIVERSITARIO CONTRA EL CANCER U A N L

Survey for the detection of COVID-19 in patients of the University Center Against Cancer

Name:______________________________ Date: ______________

Please answer the following questions			
**Have you had direct contact with any confirmed or symptomatic COVID-19 patients in the last 7 days?**	**YES**	**NO**	**1**
**Have you traveled outside the city of Monterrey in the last 15 days?**	**YES**	**NO**	**1**
**Do you have or have you had fever greater than 38ºC in the last 7 days?**	**YES**	**NO**	**3**
**Do you have a dry and persistent cough?**	**YES**	**NO**	**3**
**Do you feel short of breath?**	**YES**	**NO**	**3**
**Have you noticed a loss of taste and smell towards food?**	**YES**	**NO**	**3**
**Do you have any of the following symptoms?**<br>1.-Headache<br>3.- Nasal Runoff<br>2.- Diarrhea<br>4.- Joint or muscle pain<br>5.-Chest Pain<br>6.- Redness and itching of the Eyes<br>1 point	**YES**	**NO**	**1**
**TOTAL**			

IF YOU ADD IT TO POINTS ABOVE 7: PLEASE TRANSFER THE PATIENT TO THE COVID MODULE TO REQUEST COVID-19 PCR TEST

Laboratorio de Investigación Básica - Clínica (LIBAC)
Centro Universitario Contra el Cáncer (CUCC), Servicio de Oncología, Edificio 1, tercer piso.
Hospital Universitario "Dr. José Eleuterio González" Av. Madero y Gonzalitos S/N. Mitras Centro, Monterrey, N.L.
C.P. 64460, México. Conmutador: +52(81) 8333 – 8111 y 12, Ext: *1300, 1301, 1307, 1307, 1309 y 1310*

Figure S1

RECOMMENDATIONS FOR PATIENTS UNDER POTENTIALLY IMMUNOSUPPRESSIVE TREATMENT AND FOR THEIR FAMILY MEMBERS

- Wash your hands frequently with soap and water for at least 20 seconds. Use a hand sanitizer that contains at least 60% alcohol if soap and water are not available
- Avoid physical contact typical of the usual activity (greetings with kisses, shaking hands, hugs ...)
- Avoid spaces where there are many people (buses, metro, cinemas, and crowds of people in general)
- Avoid contact and keep distances (two meters) with people with colds, fever, cough, etc.
- Using tissues or coughing on the inside of the forearm.
- Avoid touching your mouth, eyes and nose.
- Frequent cleaning of common contact surfaces around the patient.
- The use of a mask is recommended for patients with suspicious symptoms.
- It is important that when you go for a consultation, you are accompanied only by one person.
- Always follow the instructions of your doctor and clarify any doubts in the consultation.

WHICH PEOPLE ARE MOST LIKELY OF DEVELOPING A SERIOUS ILLNESS?

Older people and those with medical conditions such as high blood pressure, heart problems, cancer, or diabetes.

IS THERE ANY VACCINE?

There is currently no vaccine that protects against COVID-19. The best way to prevent infection is to avoid close contact with sick people and wash your hands frequently.

IS THERE A TREATMENT?

There is no specific antiviral treatment for COVID-19. People with COVID-19 can seek medical attention to help relieve symptoms.

Referencias:

Organización Mundial de la Salud (OMS)
Sociedad Española de Oncología Médica
Sociedad Española de Enfermedades Infecciosas y Microbiología Médica
Centro Universitario Contra el Cáncer (CUCC)
Centrospara el Control y la Prevención de Enfermedades (CDC)

MARZO 2020

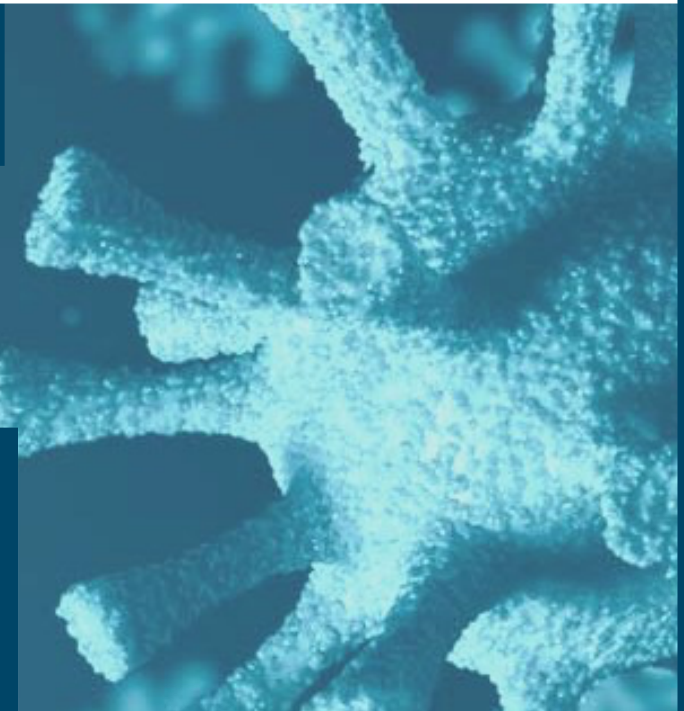

CENTRO UNIVERSITARIO CONTRA EL CANCER
U A N L

RECOMMENDATIONS ON CORONAVIRUS INFECTION (COVID-19) FOR THE CANCER PATIENT

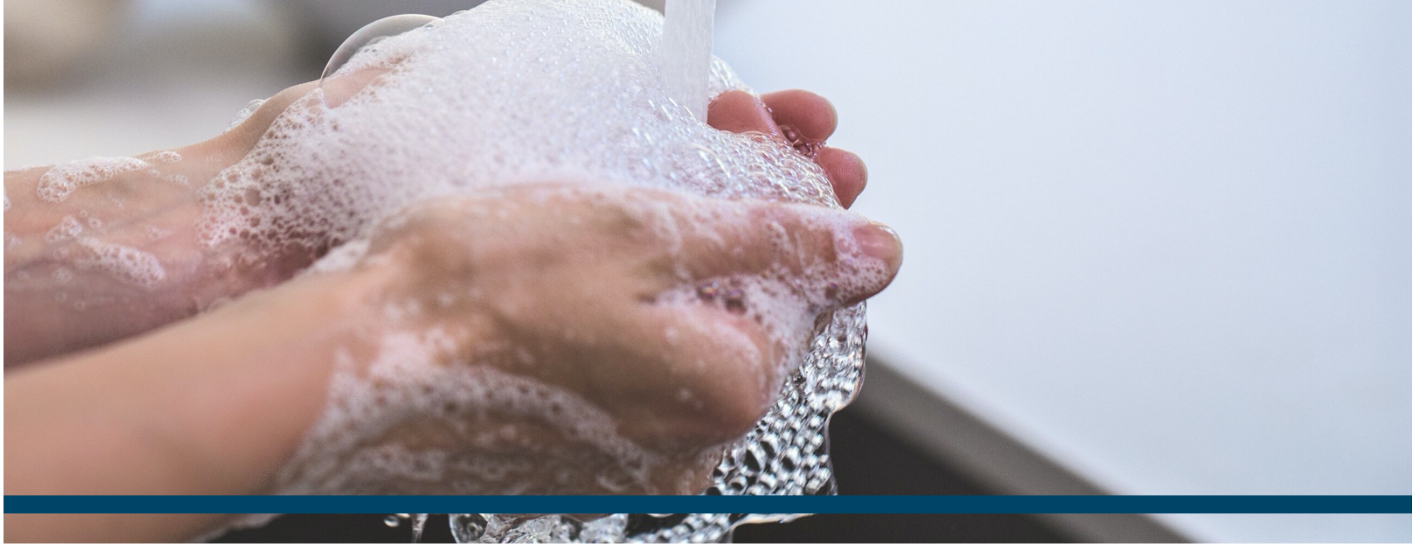

What is coronavirus disease 2019 (COVID-19)?

The coronavirus disease 2019 (COVID-19) is a respiratory condition that can spread from person to person. The virus that causes COVID-19 is a new coronavirus (SARS-CoV-2) that was first identified in Wuhan, China.

HOW COVID 19 SPREADS?

The virus is believed to spread primarily from person to person.

- Between people who have close contact with each other (2 meters apart). Through respiratory droplets that are produced when an infected person coughs or sneezes.
- It could be possible for a person to become infected by touching a surface or object that has the virus on it and then touching their mouth, nose, or possibly their eyes.

What are the symptoms of COVID-19 infection?

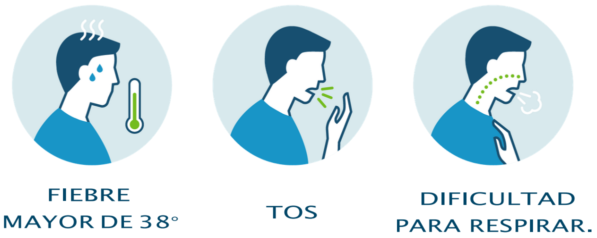

People with a fever, cough, and shortness of breath should seek medical attention. If you have these symptoms, seek medical advice quickly, as it could be due to a respiratory infection or other serious condition.

What recommendations should I follow if I have cancer?

Although so far there is no evidence of the behavior of COVID-19 in cancer patients, it is possible that patients undergoing immunosuppressive cancer treatment (such as chemotherapy) may have a higher susceptibility and a higher risk of complications than the general population. There is no evidence that COVID-19 worsens the tumor pathology itself and, in many cases, it has a mild clinical course. However, in cancer patients it could be associated with a more severe clinical picture, the severity of which could be increased if other factors such as advanced age or the presence of other comorbidities (additional or coexisting diseases) are associated.

The main cancer treatment associated with immunosuppression (lowering their defenses) is chemotherapy, therefore, in patients under active treatment, extreme precautions should be taken to avoid contagion.

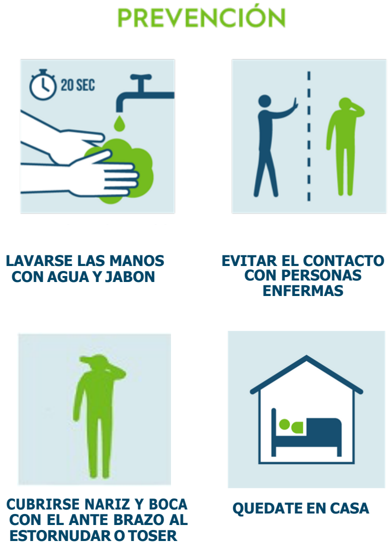

Supplement: Supplementary file 1 [file healthcare-10-00462-s001.zip › healthcare-1577551-supplementary.pdf]
